# A process-based competence evaluation for evidence-based dissemination (PROCEED): a study protocol

**DOI:** 10.1186/s40359-025-03228-4

**Published:** 2025-08-13

**Authors:** Simone Gorinelli, Essi Sairanen, Stefan G. Hofmann, Joseph Ciarrochi, Martti T. Tuomisto, Katariina Keinonen

**Affiliations:** 1 University of Jyväskylä, Jyväskylä, Finland; 2 Karlstad University, Karlstad, Sweden; 3 University of Marburg, Marburg, Germany; 4 Australian Catholic University, Sydney, Australia; 5 Tampere University, Tampere, Finland

**Keywords:** Therapist competence, Psychotherapy training, Process-based therapy

## Abstract

**Background:**

Despite extensive training to ensure high competence among psychologists and psychotherapists, scientific evidence directly linking therapist competence to treatment success remains limited. Existing competency evaluation methods often rely on subjective self-assessments or external ratings of natural therapy sessions, which are influenced by client characteristics and session context. Grounded in process-based therapy (PBT), this study aims to develop and validate a novel video-based tool (process-based competence task, PBCT) for assessing psychotherapeutic competencies.

**Method:**

The validation of the tool will consist of two phases to ensure sensitivity to previous training and responsiveness to further training. First, sensitivity of the PBCT to prior training will be assessed by comparing the performance in the task in three groups (*n* = 240): psychology university students, psychotherapists in training, and psychotherapists. Second, the responsiveness of the PBCT to further training will be assessed among psychotherapist trainees and psychology students after a follow-up period (*n* = 160). Finally, the study will examine the impact of therapist competence on treatment outcomes in a brief intervention provided by novice therapists (*n* = 70). Additional measures will include traditional self-evaluations of competence, clinical confidence, and prior training and experience. Clients in the intervention study will be assessed using various process and symptom measures.

**Discussion:**

This study aims to develop and validate the PBCT, a novel tool for assessing therapist competence. The PBCT is designed to reflect clinical skill development and knowledge on therapeutic change processes. It is expected to be sensitive to prior training and clinical experience, responsive to further training, and capable of detecting competence-outcome associations. The results are anticipated to contribute to intervention and dissemination science by providing empirical evidence on the importance of therapist training and competence.

**Trial registration:**

The study is registered at: https://doi.org/10.17605/OSF.IO/B3A7D.

## Background

The global prevalence of mental health problems is high, with almost 30% of adults experiencing a common mental health problem in their lifetime [23, 36]. While evidence-based interventions exist, there is a gap between theory and practice,evidence-based treatments are not systematically utilized and offered to individuals who need them [20, 21]. The “research-practitioner gap” highlights a critical need for developing more effective approaches of disseminating intervention science. This need has led to a shift toward a process-based approach to psychological research, which departs from the traditional approach that focuses on specific protocols tied to specific diagnoses [14]. Critical problems in the protocols-for-syndrome approach include the high comorbidity and low clinical utility of psychiatric diagnoses on the one hand and the need to master a significant amount of protocol-based treatment models to offer diagnosis-specific, evidence-based treatments on the other hand. Recognizing these problems has moved the focus of intervention science towards investigating the fundamental core mechanisms that drive changes in treatment outcomes across various forms of therapy (e.g., [16, 19]).

This shift from protocols to processes has given rise to an evolving framework in intervention science known as process-based therapy (PBT; [17]). PBT aims to organize and compress the available data to improve training and clinical practices by focusing on processes of therapeutic change that have been confirmed in mediational research and that underlie effective treatments [13]. A process-based approach to disseminating intervention science centers around enhancing clinicians’ skillfulness in identifying individual treatment targets and the dynamic networks of processes that they form and delivering evidence-based procedures that target these specific change processes, instead of delivering predetermined treatment packages that target a syndrome. The PBT approach to training can bridge the gap between research and practice and improve patient care by making evidence-based interventions easier to train and more effective to apply in clinical practice.

To generate scientific data on the effectiveness of any approach to dissemination and training of intervention skills, the key therapist competencies must first be operationalized (e.g., [18]). PBT competencies have been described in various publications (e.g., [13]). However, scientific methods for measuring and evaluating therapist competencies are still lacking. Previous attempts to establish evidence-based psychotherapy training practices have been hindered by research literature that suggests that therapist competence, i.e., the therapist's overall skillfulness in applying a given therapy method is not crucial for treatment outcome (for meta-analyses, see [35, 40]).

Research on the competence-outcome association has been neglected for the past decade. This is concerning as the existing meta-analyses have concluded that competence does not play an essential role in treatment outcomes [35, 40]. Among individual studies, authors have concluded that therapist competence is not associated with treatment outcomes for anxiety disorders, depression [5], or panic disorder [4]. In addition, a study focused on tracking the progress of psychologists receiving doctoral-level psychotherapy training found that the magnitude and speed of change observed in clients’ well-being were the same, or smaller/slower, at later stages of training [7]. The current evidence contradicts the rationale for the advanced, extensive, and expensive training required for mental health professionals. However, many of the evaluation methods employed in previous literature have not fulfilled the criteria for competence evaluation first proposed by Perepletchikova and Kazdin [33] and later emphasized by other researchers in the field (e.g., [27, 34]). It is widely agreed that therapist skillfulness should be evaluated using methods that are a) sensitive to differences among novices and highly experienced therapists and b) responsive to further training.

To date, the validity of many competence measures has not been systematically evaluated and reported. The problem may have been exacerbated by the current requirement to report an evaluation of therapists’ adherence and competence in randomized controlled trials as part of research integrity reporting. The focus has been on validating the design of the intervention under examination in a given trial, instead of the sensitivity of the method used for evaluating competence. This may have yielded overly optimistic ratings of competence. For example, some studies have reported “very good” or “excellent” competence for 92% of novice therapists [10]. Another common issue is the considerable variation in the trial-specific conceptualization and rating procedures. As research on competence-outcome effects is arguably lacking, the comparability of results is a crucial consideration. Variation in competence evaluation stemming from confounding factors, such as alliance or the nature and severity of the participant’s symptoms, can also influence or obscure the relationship between competence and outcomes [40]. This may be a severe shortcoming, as it directly undermines the importance of advanced training for mental health professionals. Overall, previous null results of the association between competence and outcome may reflect methodological issues, such as poor conceptualization, rating competence in settings that include multiple sources of variation, and insufficient attention to the sensitivity of existing competence measures. This calls for revisiting the research on therapist competence with adequate precision. A process-based therapy approach, which focuses on core clinical competencies in targeting empirically established change processes, can guide the development of novel competence evaluation methods.

The development of therapists’ clinical skills has been construed as a continuous process that builds on basic skills and becomes more refined and flexible with repetition and learning. Building clinical skills involves several increasingly complex phases: content knowledge (“knows”), competency (“knows how”), performance (“shows how”), and action (“does”; [30]). As simulated and natural sessions that would allow “showing how” or “doing” therapy include many sources of variation, standardized evaluation methods can be overly complicated to create. Many previous methods have relied on external observer ratings or self-rated evaluations, which can be highly subjective. However, evidence suggests that a high perceptual ability to identify clinically appropriate behaviors and discriminate between effective and ineffective behavior indicates expert-level skillfulness in the context of therapy and medicine [8, 26], which suggests that evaluating the degree to which a therapist “knows how” can be sufficient.

This project proposes a performance-based approach to competence evaluation that focuses on the know-how of participants using an online task to identify clinically relevant behaviors and competencies in video-recorded, simulated therapeutic discussions. Some preliminary findings suggest that this approach has been successful in discriminating between previous training levels, and is responsive to further training in two different therapy approaches: acceptance and commitment therapy (ACT; [27]) and dialectical behavioral therapy (DBT [41],). Both ACT and DBT can be seen as forms of process-based therapies, where the focus of the intervention is on identifying individual intervention needs and applying specific techniques to target underlying processes of change. However, competence evaluation approaches must allow comprehensive utilization across various therapies to serve the renewal of dissemination science. This is possible if a new competence evaluation method is developed based on the theoretical frame of process-based therapy (PBT, [15]) for evaluating competencies in various empirically established approaches.

Therefore, previous issues can be addressed by a) focusing on the sensitivity of the method for evaluating competence and validating the method scientifically before further analyses, b) utilizing a process-based approach to measuring competence that allows competence evaluation in training across evidence based methods, and c) removing competence evaluation from clinical contexts to decrease the interference of other processes (e.g., client behavior and the nature and severity of their problem, personal characteristics, and life-events of the therapist).

## Methods

### Design

The current protocol describes a four-year (2024–2028) research project funded by the Research Council of Finland (project number 362855). The research protocol has been reviewed and approved by the Human Sciences Ethics Committee of the University of Jyväskylä on 16.12.2024 (Ref. 1566/13.00.04.00/2024), in accordance with the Declaration of Helsinki.

### Research objectives and tasks

By applying the approach described above, the current research aims to bridge the gap from theory to practice by operationalizing therapist competence based on understanding clinical skills development and the cutting-edge science of change processes in psychotherapy. In essence, the current study aims to develop and validate the process-based competence task (PBCT) and, thus, to respond to the challenge of demonstrating a) the efficacy of therapist training efforts and b) the importance of therapist competence in successful treatment delivery.

The PBCT is expected to show sensitivity to previous training and experience and responsiveness to further training. It is also expected that the novel approach to competence will detect the competence-outcome relationship. These findings would advance the field of intervention and dissemination science by providing empirical evidence on the importance of sufficient training and skillfulness. This would lead to further innovative research opportunities in three intertwining areas that benefit from measuring competence: 1) identifying therapeutic change processes, 2) increasing evidence-based treatments’ effectiveness, and 3) building a scientific framework for evidence-based training and dissemination.

The current research will progress in three subsequent sub-studies that build upon one another. The study aims to meet the following research goals in these three sub-studies:

- *The aim of Study 1* is to develop the PBCT and validate sensitivity to therapist experience. Research questions under Study 1 are: a) Does the PBCT show sensitivity to previous experience and training? b) Are therapists’ formal and informal training, work-related variables (e.g., work settings), and self-reported attributes related to competence and confidence associated with performance in the PBCT? This validation stage will be deemed successful if performance in the PBCT is associated with the level of training.
- *The aim of Study 2* is to continue the validation of the PBCT by ensuring responsiveness to further training. The second validation stage will be deemed successful if performance in the PBCT is improved after additional training for master’s students of psychology and psychotherapist trainees. In addition, the association between therapist characteristics and competence development are analyzed. Research questions under Study 2 are: a) Does the PBCT detect increases in competence during one year of psychotherapist training or brief psychology master’s program training? b) Are therapists’ formal and informal training, work-related variables (e.g., work settings), and self-reported attributes related to competence and confidence associated with performance in the PBCT?
- *The aim of Study 3* is to provide evidence of the importance of therapist competence for treatment outcomes in a brief intervention for enhancing mood and wellbeing delivered by novice therapists. In addition, competence development during brief training and intervention delivery is examined. Research questions under Study 3 are: a) Does the PBCT detect increases in novice therapists' competence after brief training or during the intervention? b) Is competence associated with the outcome of a brief intervention for mood problems provided by novice therapists? c) Can individual differences in the initial level of competence and changes in competence during training or delivery of the intervention be identified and modeled to improve training practices?

## Protocol

### Study 1

#### Design and setting of the study

The PBCT sensitivity to previous training will be validated by comparing performance in the task between three populations with different levels of previous training and clinical experience, i.e., psychology master's degree students, psychotherapy trainees, and psychotherapists.

#### Participants

Participants in the validation study (*N* = 240) are 1) master’s level psychology students (novice therapists; *n* = 80), 2) students in training to become psychotherapists (therapist trainees; *n* = 80) and 3) practicing psychotherapists with full four years of training (experienced therapists; *n* = 80). All master’s students in psychology at the University of Jyväskylä, psychotherapist trainees and psychotherapists in Finland who have been trained in CBT are eligible to participate in the study. Some training in CBT will be required due to the emphasis on CBT methods in the design of the PBCT selection (see below). There are no other exclusion criteria for the student and therapist participants. An additional sample of bachelor’s level students of psychology will be recruited to participate for the purpose of examining the test–retest reliability of the PBCT.

#### Measures

##### **The process-based competence task (PBCT)**

Scripts for a total of 24 brief simulations (approx. 2–3 min., total duration of the task approx. 60–75 min.) have been created. The simulated conversations are focused on individual treatment targets (case vignettes) and use of specific intervention competencies targeting processes of change established in mediation research. Existing scripts available in the clinical literature (e.g., manuals and handbooks on CBT and PB-CBT) have been utilized to write the scripts in English. Simulated conversations have been developed in English by the Authors, translated to Finnish, and video recorded using local actors in Finnish. The competencies included in the recordings consist of delivering evidence-based CBT methods targeting specific processes of change, e.g., emotion regulation, exposure strategies, behavioral activation, enhancing interpersonal skills, modifying core beliefs, present-moment awareness, acceptance, and motivational strategies (Hayes & Hofmann, 2018, [22]). In its final form, the PBCT will be completed as an online task that asks participants to identify key elements in each simulation, including the dimensions of psychological functioning being targeted by the therapists, the core clinical competency used by the therapist, the function of the therapist’s behavior. Participants will also rate the quality of the therapeutic relationship or interaction. Videos will be presented in a randomized order to control for potential order effect such as learning, fatigue, or other biases. A scoring key will be created to allow scoring each subscale and total score. The correct answer for each identification and rating item will be based on expert consensus between the Authors, who will use external raters and AI language models trained to detect and code relevant features of simulation scripts to support their decision-making as needed.

Other measures will involve demographic and professional background information, along with self-report measures and validated questionnaires.

Background information will include demographic variables (option categorized, e.g., age group, gender, primary field of work, marital status etc.), previous formal training (e.g., degrees additional training, workshops), and previous informal training (e.g., online courses, books).

Self-report measures include surveys of competence, clinical confidence, experiential avoidance, and psychological flexibility. Skillfulness, familiarity with CBT and PBT, and clinical confidence will be measured with self-rate ad-hoc questions. Professional wellbeing (i.e., compassion satisfaction and compassion fatigue) will be measured using the Professional Quality of Life Scale (ProQOL; [39]), while The Comprehensive assessment of Acceptance and Commitment Therapy Processes (CompACT-10 [12],) and The Acceptance & Action Questionnaire (AAQ-2 [3],) will be used to measure psychological flexibility and experiential avoidance, respectively. A self-evaluation of competences scale will also be developed and used to establish self-perceived experience of skillfulness in the domains evaluated with the PBCT. The self-evaluation scale will be developed by incorporating and adapting relevant items from the Judy Beck Cognitive Therapy Rating Scale – Revised (CTRS-R [2, 42],), the Assessment of Core CBT skills (ACCS [31],), and other available measures to evaluate CBT skill competence.

#### Procedure

Participants are recruited a) as part of teaching practices of bachelor’s and master’s level psychology students in the University of Jyväskylä (JYU), and via email lists and social media channels of b) CBT psychotherapist trainees in national CBT training programs, and 3) CBT psychotherapists working in major CBT psychotherapy centers and working as private practitioners. JYU students are invited to complete the online survey during lectures on clinical psychology or psychosocial interventions and psychotherapies. Students will also be recruited via email lists for relevant courses. Students may decline to participate without any impact to their studies. Therapist trainees are contacted in collaboration with Finnish CBT psychotherapist training providers, and Finnish CBT associations. Fully trained psychotherapists are also contacted in collaboration with national CBT associations and via psychotherapy centers that provide facilities for CBT psychotherapists for clinical work across Finland. In addition to email lists, social media channels (e.g., Whatsapp, Facebook, LinkedIn and Instagram) that are aimed for psychotherapist trainees or psychotherapists are used to inform potential participants about the study. All communications will include information on eligibility criteria of having had some training in CBT. The emails and social media posts contain a brief research announcement and an invitation to participate in the research. All communications include a link that will take potential participants to the JYU website, where the complete information letter included in the informed consent is publicly available in REDCap, a secure, web-based application used for collecting and managing electronic survey data. Potential participants can access the survey that include the informed consent after reading the information letter on JYU website. After consent is obtained, participants are immediately able to respond to the survey.

Students visit the REDCap survey during a lecture or independently, while therapists in training and expert therapists access the survey independently through the survey link. Participants are firstly presented with demographic and background information aimed at exploring demographic variables, previous training, and self-evaluation of clinical competence. Before starting the PBCT, participants complete a self-assessment of their clinical competencies and other questionnaires. They will then complete the PBCT by watching several short videos and answering a few questions after each video. Upon completing the survey, participants will receive automated feedback based on their self-assessment of competencies and measures of professional quality of life, psychological flexibility and experiential avoidance. This feedback is designed to address any potential discomfort that may arise, particularly if they perceive their competence to be low.

#### Analysis

The analysis strategy for Study 1 will focus on examining between-group differences, emphasizing mean differences and exploring associations between self-reported and tested competence and related professional qualities. Power analyses to determine appropriate sample sizes were calculated using an online tool available at https://www.danielsoper.com/statcalc/. For Study 1, a minimum sample size per group was determined to be 64 for a two-tailed t-test when the anticipated effect size (d) was set to 0.50, and the desired power level was set to 0.80. To anticipate drop-out during data collection, the sample size was set to 80 participants per group.

### Study 2

#### Design and setting of the study

The psychotherapist trainees participating in Study 1 are invited to complete the PBCT and questionnaires after a follow-up period of one year to examine the sensitivity of the PBCT to further training and the development of competence during advanced training. In addition, the same psychology students participating in Study 1 are invited to complete again the PBCT and questionnaires after completing masters-level courses on clinical CBT to examine sensitivity to brief further training. Follow-up period for students is approximately six months. To account for the measurement effect, bachelor’s level psychology students not receiving additional training are recruited to complete the PBCT at two time points during three months.

#### Participants

Participants (*N* = 240) are 1) master’s level psychology students participating to Study 1 (novice therapists; *n* = 80), 2) students in training to become CBT psychotherapists participating to Study 1 (therapist trainees; *n* = 80) and 3) bachelor’s level psychology students (control participants; *n* = 80).

#### Measures

Measures include the same background information, self-rate questions and self-evaluation scale of competence and the PBCT as used in Study 1. Additionally, Study 2 will include a self-evaluation of improvement in competence after the previous survey.

#### Procedure

Students will be invited to complete the REDCap survey approximately six months after attending the first lecture. Students may participate during a lecture or independently. Therapists in training access the survey independently through the survey link that is sent them by email approximately one year after responding to the first survey.

#### Analysis

The analyses will concentrate on within-group mean changes, utilizing person-centered analysis strategies to identify individual trajectories of change in competence. The same power analysis for Study 1 was used for Study 2, where a minimum sample size per group was determined to be 64 for a two-tailed t-test when the anticipated effect size (d) was set to 0.50, and the desired power level was set to 0.80. To anticipate drop-out during data collection, the sample size was set to 80 participants per group.

### Study 3

#### Design and setting of the study

Master’s level psychology students will receive a brief training on process-based CBT training, after which they deliver a short intervention to clients. Master’s level psychology students receive three days of training on a) building a functional case conceptualization to identify individual treatment targets, and b) using process-based CBT processes of therapeutic change. The training and provision of interventions are built in the mandatory studies offered to students completing a master’s degree in psychology. This novel training and related interventions serve as one of several modes of study for the students. Weekly supervision and peer-supervision are included in the curriculum for this mode of study on the course.

Second, the students will deliver the six-session intervention to two client participants. Interventions will be delivered in three waves. Students will receive weekly supervision from clinical psychologists and psychotherapist and attend weekly peer-supervision.

#### Participants

Participants are master’s level psychology students (*n* = 70) and voluntary individuals (*N* = 140) who seek support for common psychological problems (e.g., low mood, anxiety, psychological distress).

The inclusion criteria for client participants are: 1) willingness to participate in an intervention aimed at increasing psychological wellbeing that is delivered by a psychology student, 2) having experienced one or several common problems of psychological wellbeing, i.e., stress, low mood or anxiety related problems, 3) willingness and ability to participate in a study that requires visits to the Psychotherapy Teaching and Research Clinic at JYU and responding to online surveys. The exclusion criteria will be: 1) severe psychological ill-being that requires assessment and treatment by a mental health professional, 2) psychological symptoms that significantly affect daily functioning, 3) ongoing psychological treatment or psychotherapy, 4) changes in medication prescribed for psychological symptoms during past 4 months, 5) alcohol or drug addiction, 6) self-injurious behaviors, or 7) current suicidal thoughts.

#### Measures

Measures include the same background information, self-rate questions and self-evaluation scale of competence and the PBCT as used in Study 1. In addition, students’ therapy sessions will be recorded using the MyJYU AI transcript service, which automatically transcribes audio recordings into text. Text transcripts will be used as data to facilitate the analysis of session content.

Client participants complete background questionnaire information such as demographic variables (e.g., (options categorized, e.g., age group, gender, marital status, working status), expectations and attitudes toward the intervention provided by students (e.g., beliefs of usefulness, expectations of benefits, expectations of student therapist competence), and health-related demographic variables (options categorized, e.g., previous use of psychological support services, current use of medication for somatic or psychiatric conditions, current somatic health issues, self-evaluated current health).

Additionally, client participants in WP3 will complete several questionnaires to assess their situation before the intervention, during the intervention (after session 3) and after the intervention. Shorter versions were selected to minimize response fatigue, while multiple questionnaires were chosen to capture a range of psychological processes. Measuring various aspects helps address co-occurring indicators in the client’s situation and identify key psychological processes of change that could lead to better outcomes in process-based interventions. The aim is to measure both symptoms and processes to explore different psychological dimensions and therapeutic targets, providing a more comprehensive understanding and assessment of the client, while keeping the measures limited in length to avoid fatigue. The process-based assessment tool (PBAT; [6]) will be used to measure processes of change, outcome, compassion and therapeutic alliance, the Comprehensive assessment of Acceptance and Commitment Therapy Processes (CompACT-10 [12],) to measure psychological flexibility, The Acceptance & Action Questionnaire (AAQ-2 [3],) to measure experiential avoidance, and the Cognitive Fusion Questionnaire (CFQ-13 [11],) to measure cognitive processes and fusion with thoughts. The Depression Anxiety Stress Scales – Short Form (DASS21 [28],) will be used to measure levels of mood and stress-related difficulties, the Patient Health Questionnaire (PHQ-9 [25],) to measure levels of mood-related difficulties, the Generalized Anxiety Disorder scale (GAD-7 [38],) to measure levels of anxiety-related difficulties, while the Shirom-Melamed Burnout Measure short version (SMBM-6 [1],) and the Dimensions of Anger Reactions questionnaire (DAR-5 [9],) to measure experiences of burnout and experience of anger respectively. The Mental Health Continuum Short Form (MHC-SF [24],) will be used to measure wellbeing, the Adult State Hope Scale (ASHS [37],) to measure sense of hope, including their goal-directed motivation and ability to find strategies to achieve goals, the State Self-Compassion Scale (SSCS-L [32],) to measure self-compassion and its subcomponents, and the Brunnsviken Brief Quality of Life Scale (BBQ [29],) to measure life satisfaction across six-life areas. Finally, life quality and physical health will be measured with an ad-hoc 1-item VAS scale.

#### Procedure

Recruitment for Study 3 is conducted in two arms: a) student participants are recruited within the context of teaching offered at the department of psychology at JYU, and b) client participants are recruited via advertisements and announcements circulated via email lists for mental health providers, publicly in social media and in the calls for study participants published on the JYU website.

Students are invited to enroll in a specific study mode of a course focused on delivering psychological interventions. This course is part of mandatory studies for a master’s degree in psychology. The current study provides one of the alternative modes offered on the course. The course content does not differ from general teaching practices, apart from completing the measures and other research related tasks.

Client participants are invited to take part in the study using targeted calls for participants that are circulated among local mental health providers and private practitioners who may identify persons who might be interested to participate in the study. Client participation is also invited by open calls in the JYU website for calls for participants and by a notice on the website of the Psychotherapy Research and Teaching Clinic at JYU. In addition, social media notifications are sent out in online groups and communities for mental health professionals and posted on public social media accounts for the project. These recruitment methods are aimed at identifying possible participants who are motivated in participating in a psychological intervention, but who do not have acute need for professional evaluation and treatment. All client participants must meet the inclusion and exclusion criteria designed to ensure client participants are fully aware that the interventions are provided by students practicing the provision of interventions and that participation does not substitute psychological treatment in situations where an individual feels they are in need of professional help. The self-report-based suitability assessment is accompanied by careful review of self-report scales of the level of psychological ill-being that all participants fulfill before initiating the interventions. In case a client participant reports severe symptoms that indicate the need for a professional evaluation and treatment planning, the participant will be excluded from the study and instructed to contact regional mental health services. To enroll in the study, participants respond to the initial enrollment survey that includes eligibility criteria and providing informed consent if eligibility criteria are met. After obtaining a consent, the research team view participants’ information and assign participants to student therapists. Students contact clients using their university email to schedule the first meeting that consist of an initial interview. Students are instructed to use email only to set up the initial meeting time and to provide standardized instructions on next steps. A research assistant is hired to manage all further communication, including handling cancellations. After contacting the client and making sure that consent is obtained, students schedule a time for the first session.

Before starting each session, students log in their MyJYU application to audio record the sessions, which are safely stored on a secure JYU database. Clients are asked to complete a survey containing questionnaires on psychological well-being, flexibility, symptomatology, and daily functioning after giving consent and during the week before their session (more details in measurement section). They are asked to complete the same survey again after the third session of the six-session intervention and at the end of the intervention.

Students conduct the PBCT and other measures at four time-points: one month before the training begins, immediately before the training, after training, and after the interventions have been delivered. This allows for the evaluation of the test–retest properties of the PBCT, and for establishing a baseline of variation in competence in the absence of training. Client participants are asked to complete outcome and process questionnaires at three time-points: recruitment, after session three, and after the intervention.

#### Analysis

The analyses for Study 3 will focus on the relationship between treatment outcomes and competence levels using multiple regression analyses, while also examining within-group changes in competence among students during baseline, training, and intervention measures. For Study 3, a minimum sample size for client participants was 118 for multiple regression analysis with up to ten predictors in the model to examine multiple change predictors. The anticipated effect size (f2) was set to 0.15, and the desired power level was set to 0.80. To anticipate drop-out, the sample size of client participants was set to 140 (70 student therapists with two clients each).

**Fig. 1:**
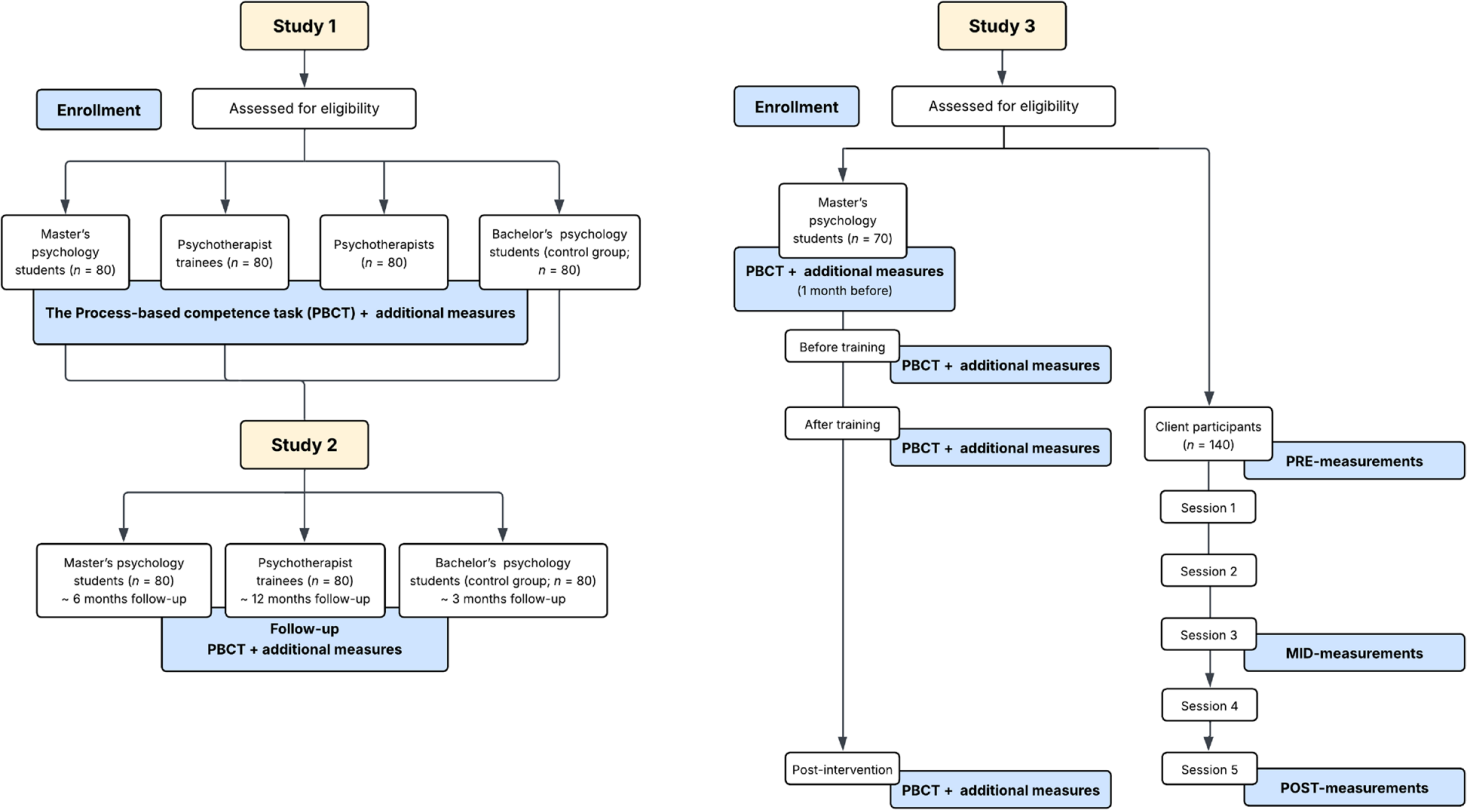
Design and structure of the studies

The overall design of the studies is illustrated in Fig. 1 below.

## Discussion

The current study represents a novel effort to operationalize therapist competence through the development and validation of a video-based assessment task grounded in process-based therapy (PBT). While special attention has been paid to methodological rigor, several practical and operational considerations must be considered when implementing its procedures to minimize risks that could negatively affect generalizability and further implementation of key findings.

First, the research will be conducted in three subsequent sub-studies, from initial development and validation to longitudinal validation, and finally integration into training and intervention. The multi-phase design is vulnerable to unexpected problems at early stages of the research that may prevent progressing to subsequent phases. To minimize delays in proceeding thru the planned sub-studies, actions will be taken to ensure participant retention, especially across the one-year follow-up in Study 2, participant attrition due to workload and time-requirement for participants. These actions include the use of automated reminders, providing feedback on self-evaluations related to professional factors and wellbeing to participants, and incorporating the study into course structures. Second, while the format of the PBCT offers a standardized approach to evaluating therapist competence, there are limitations in assessing the “know-how” of participants without observing actual therapist behavior. A standardized design helps to limit the effect of factors that influence therapist behavior in clinical settings (such as client behavior or nature of client’s problems) and act as confounding factors in measuring competence. However, it also limits the range and variance of therapeutic interactions that are present in the standardized material and all possible aspects of competent therapist behavior will not be included in the scope the current task. This will require clear and precise description of the definition of competence in the current studies and it will also have to be considered when generalizing the results. A third issue concerns the psychometric properties of the PBCT. As with any novel assessment, it is critical to ensure cultural and linguistic validity. In the current research, the tool has been developed in English in collaboration with Authors and translated to Finnish for Finnish participants. Subsequent use in international contexts will require ensuring that rating instructions, item wording, and clinical concepts translate effectively across languages and therapeutic cultures. Additionally, the use of university students as novice therapists delivering interventions in Study 3 reflects both a strength and limitation. On the one hand, it provides a controlled environment for examining the PBCT properties in the context of training, but on the other, it limits generalizability to broader training and professional contexts. Also, reliance on self-reported inclusion/exclusion criteria by clients introduces potential variability in symptom severity and motivation levels, which may affect the interpretation of competence-outcome relationships in the intervention study. Finally, ethical and educational responsibilities must be considered. All participants need to be carefully instructed and debriefed when completing the novel PBCT to ensure participation in the study will not have a demoralizing effect for students, trainees or experienced clinicians. Likewise, client participants must be informed about protocols for symptom monitoring and prompt referral in cases of significant distress to ensure that participation is safe and ethically sound.

In summary, while the PROCEED study offers a promising direction for competency-based training and dissemination science, its implementation must balance scientific rigor with pragmatic constraints. Addressing these issues transparently will strengthen the interpretability, reproducibility, and eventual impact of the findings.

## Data Availability

No datasets were generated or analysed during the current study.

## Abbreviations

AAQ-2: The Acceptance & Action Questionnaire
ACCS: Assessment of Core CBT skills
ACT: Acceptance and commitment therapy
ASHS: Adult State Hope Scale
BBQ: Brunnsviken Brief Quality of Life Scale
CBT: Cognitive behavioral therapy
CFQ-13: Cognitive Fusion Questionnaire
CompACT-10: The Comprehensive assessment of Acceptance and Commitment Therapy Processes
CTRS-R: Judy Beck Cognitive Therapy Rating Scale – Revised
DAR-5: Dimensions of Anger Reactions questionnaire
DASS21: Depression Anxiety Stress Scales – Short Form
DBT: Dialectical behavioral therapy
GAD-7: Generalized Anxiety Disorder scale
JYU: University of Jyväskylä
MHC-SF: The Mental Health Continuum Short Form
PBAT: The process-based assessment tool
PBCT: Process-based competence task
PBT: Process-based therapy
PHQ-9: Patient Health Questionnaire
ProQOL: Professional Quality of Life Scale
SMBM-6: Shirom-Melamed Burnout Measure short version
SSCS-L: State Self-Compassion Scale
VAS: Visual Analogue Scale
